# Editorial: Trends in neurocritical care

**DOI:** 10.3389/fneur.2024.1403510

**Published:** 2024-05-01

**Authors:** Po-Huang Chen, Hong-Jie Jhou, Cho-Hao Lee, Shey-Lin Wu

**Affiliations:** ^1^Division of Hematology and Oncology Medicine, Department of Internal Medicine, Tri-Service General Hospital, National Defense Medical Center, Taipei, Taiwan; ^2^Department of Neurology, Changhua Christian Hospital, Changhua, Taiwan; ^3^Department of Neurology, Chang Bing Show Chwan Memorial Hospital, Changhua, Taiwan; ^4^Department of Electrical Engineering, National Changhua University of Education, Changhua, Taiwan

**Keywords:** neurocritical care, movement disorder emergency, subarachnoid hemorrhage, ischemic stroke, stroke

The growing need for neurocritical care underscores the profound impact of neurocritical illnesses and their associated high rates of morbidity and mortality (1). As an evolving subspecialty, neurocritical care is dedicated to delivering targeted care for neurological emergencies, aiming to rapidly address acute neurological issues to enhance patient survival and recovery. Quick and accurate identification of these conditions, followed by immediate treatment, is crucial, significantly affecting patient outcomes. The specialty also navigates complex challenges such as predicting recovery of consciousness and determining long-term functional outcomes, in addition to ethical considerations regarding life-support measures. These complexities highlight the urgent requirement for progress in neurocritical care. The insightful research contributions we have received, including studies on the critical role of serum sodium management in conditions like spontaneous subarachnoid hemorrhage (SAH) and acute ischemic stroke, and unique case reports, shed light on these challenges and emphasize the importance of meticulous patient management to improve outcomes in this delicate field.

Jin et al. examine the association between serum sodium levels and in-hospital mortality in critically ill patients with spontaneous SAH. The research aims to retrospectively analyze the correlation between serum sodium concentrations upon ICU admission, minimum serum sodium levels during ICU stay, sodium fluctuations, and the mortality of these patients. Utilizing data from the Medical Information Mart for Intensive Care IV (MIMIC-IV) database, the study employs restricted cubic splines to investigate the serum sodium-in-hospital mortality relationship and receiver operating characteristic analysis to identify the optimal sodium fluctuation cutoff for predicting mortality. The findings reveal a J-shaped relationship between initial and minimum ICU sodium levels with in-hospital mortality, highlighting that sodium fluctuations above a certain threshold are independently linked to increased mortality risks. The study underscores the importance of monitoring and managing serum sodium levels in SAH patients to improve outcomes, suggesting the need for further prospective research to validate these findings.

Yang et al. conducted a study to identify risk factors and develop prediction models for prolonged hospital stays following acute ischemic stroke, utilizing artificial neural networks (ANNs). By examining medical records from patients treated at a stroke center, the study aimed to provide accurate estimates of hospital stay duration to better understand medical expenses and plan patient discharge processes. A prolonged stay was defined as exceeding the median length of stay, with ANN models developed based on admission parameters to predict these extended durations. Through sensitivity analysis and 5-fold cross-validation, the study identified several key factors associated with prolonged stays, including severity of stroke upon admission, atrial fibrillation, thrombolytic therapy, and histories of hypertension, diabetes, and previous strokes. The ANN models demonstrated reasonable accuracy, sensitivity, and specificity in predicting prolonged hospital stays, highlighting their potential utility in clinical assessments, decision-making, and the creation of tailored medical care plans for acute ischemic stroke patients.

In the case report by Wang et al., a rare instance of progressive chorea as the manifestation in a female patient with both Moyamoya and Graves' disease is described. Chorea, a movement disorder characterized by brief, involuntary, random hyperkinetic movements, was observed alongside the unusual coexistence of these two conditions. Diagnostic imaging revealed mild to moderate hypoperfusion in specific brain regions. The patient's choreic movements showed rapid improvement following a 5-day treatment with dexamethasone, highlighting the effectiveness of steroids in such cases. This report underscores the rare but possible co-occurrence of thyrotoxicosis and Moyamoya disease, particularly in Asian female adults. It emphasizes the complex interaction between excessive thyroid hormones and ischemic changes in the brain, which can exacerbate each other's effects. The findings suggest the importance of assessing thyroid function in patients with Moyamoya disease and propose that steroid treatment could have a dual benefit by mitigating thyroid-related inflammation and modulating neurotransmitter activity to improve cerebral perfusion.

Liu et al. conducted a retrospective study to explore the association between serum sodium levels within the first 24 h of admission and all-cause mortality among critically ill patients with non-traumatic SAH, utilizing the MIMIC-IV database. The research aimed to determine whether initial serum sodium levels could predict mortality rates in the ICU and hospital settings. Through a comprehensive analysis involving multivariate Cox proportional hazard regression models and Kaplan–Meier survival curves, the study identified a J-shaped non-linear relationship between serum sodium at ICU admission and mortality rates, pinpointing an inflection point around 141 mmol/L. This suggests that both hypernatremia are associated with increased mortality risks, with a significant rise in risk observed in patients with serum sodium levels ≥145 mmol/L. The findings indicate that high serum sodium levels are significantly correlated with an increased risk of death, emphasizing the necessity for careful management of serum sodium in this patient population. The study highlights the critical importance of monitoring and potentially adjusting serum sodium levels as part of the treatment strategy for patients with non-traumatic SAH to improve outcomes.

In conclusion, the research featured in this editorial highlights the critical intersections between clinical outcomes and physiological parameters in neurocritical care. From the nuanced relationship between serum sodium levels and mortality in patients with spontaneous SAH to the identification of risk factors and predictive models for prolonged hospital stays post-acute ischemic stroke, these studies underscore the importance of early, precise management and monitoring in the neurocritical care domain. Moreover, the intriguing case of steroid-responsive acute chorea revealing the coexistence of Moyamoya and Graves' disease further illustrates the complex interplay between neurological and systemic diseases and the potential for innovative treatment approaches.

Collectively, these contributions not only advance our understanding of neurocritical illnesses but also emphasize the need for interdisciplinary approaches to diagnose, monitor, and treat these conditions effectively (2). In earlier research, we found ischemic stroke patients with high anion gaps or hyperglycemia have poorer outcomes, suggesting more factors affect prognosis (3, 4). It is advocated that artificial intelligence, specifically artificial neural networks, be employed for comprehensive patient assessments. This strategy is aimed at developing predictive models to facilitate personalized care, with the ultimate objective of reducing morbidity and mortality. As we move forward, it is imperative that we continue to refine our clinical strategies based on robust evidence, embrace technological advancements in patient care, and foster a deeper understanding of the pathophysiological mechanisms at play. The journey toward improving outcomes in neurocritical care is ongoing, and the insights gained from these studies are valuable steps in that direction, encouraging further research and innovation in this vital field of medicine.

## Author contributions

P-HC: Writing – original draft. H-JJ: Writing – original draft. C-HL: Writing – original draft. S-LW: Writing – review & editing.
